# HIV-Exposed Uninfected Infants in Zimbabwe: Insights into Health Outcomes in the Pre-Antiretroviral Therapy Era

**DOI:** 10.3389/fimmu.2016.00190

**Published:** 2016-06-06

**Authors:** Ceri Evans, Jean H. Humphrey, Robert Ntozini, Andrew J. Prendergast

**Affiliations:** ^1^Zvitambo Institute for Maternal and Child Health Research, Harare, Zimbabwe; ^2^Blizard Institute, Queen Mary University of London, London, UK; ^3^Department of International Health, Johns Hopkins Bloomberg School of Public Health, Baltimore, MD, USA

**Keywords:** HIV exposure, infant, Zimbabwe, Africa, inflammation, immune activation, breast-feeding

## Abstract

The ZVITAMBO trial recruited 14,110 mother–infant pairs to a randomized controlled trial of vitamin A between 1997 and 2000, before the availability of antiretroviral therapy for HIV prophylaxis or treatment in Zimbabwe. The HIV status of mothers and infants was well characterized through 1–2 years of follow-up, leading to the largest cohort to date of HIV-exposed uninfected (HEU) infants (*n* = 3135), with a suitable comparison group of HIV-unexposed infants (*n* = 9510). Here, we draw on 10 years of published findings from the ZVITAMBO trial. HEU infants had increased morbidity compared to HIV-unexposed infants, with 50% more hospitalizations in the neonatal period and 30% more sick clinic visits during infancy, particularly for skin infections, lower respiratory tract infections, and oral thrush. HEU children had 3.9-fold and 2.0-fold higher mortality than HIV-unexposed children during the first and second years of life, respectively, most commonly due to acute respiratory infections, diarrhea/dysentery, malnutrition, sepsis, and meningitis. Infant morbidity and mortality were strongly related to maternal HIV disease severity, and increased morbidity remained until maternal CD4 counts were >800 cells/μL. HEU infants were more likely to be premature and small-for-gestational age than HIV-unexposed infants, and had more postnatal growth failure. Here, we propose a conceptual framework to explain the increased risk of infectious morbidity, mortality, and growth failure among HEU infants, hypothesizing that immune activation and inflammation are key drivers of both infection susceptibility and growth failure. Future studies should further dissect the causes of infection susceptibility and growth failure and determine the impact of ART and cotrimoxazole on outcomes of this vulnerable group of infants in the current era.

## Introduction

Before the availability of antiretroviral therapy (ART), around a quarter of infants born to HIV-infected women in Zimbabwe acquired the infection (1) and almost two-thirds of perinatally infected children died before their second birthday (2). Coverage of effective interventions for prevention of mother-to-child transmission (PMTCT) is increasing faster than antenatal HIV prevalence is declining, meaning that fewer HIV-infected infants are born annually, but a population of HIV-exposed uninfected (HEU) infants is emerging (3). Data from several settings over the past decade suggest that HEU children have poorer health outcomes than HIV-unexposed children. However, many studies have not fully characterized maternal and infant HIV status, or have included control groups of HIV-unexposed infants who differ significantly in socioeconomic status or breast-feeding pattern, which may lead to confounding. Furthermore, modern studies are complicated by exposure to maternal and infant ART for PMTCT or cotrimoxazole prophylaxis, making the natural history of HIV exposure difficult to determine. Findings from historical cohorts with comparable control populations are necessary to understand the health outcomes of HEU infants.

Between 1997 and 2000, 14,110 mother–infant pairs were recruited to a randomized controlled trial of maternal and infant vitamin A in Zimbabwe. The Zimbabwe Vitamin A for Mothers and Babies (ZVITAMBO) trial (1) took place before the availability of ART for prophylaxis or treatment in Zimbabwe, or the recommendation to provide cotrimoxazole to HIV-exposed infants. The HIV status of mothers and infants was well characterized through 1–2 years of follow-up, leading to the largest cohort to date of HEU infants (*n* = 3135), with a suitable comparison group of HIV-unexposed infants (*n* = 9510). This Review draws on 10 years of published data from this birth cohort, which has provided some of the strongest evidence to date of the poor health outcomes of HEU infants. First, we discuss the morbidity and mortality of HEU infants and the relationship between maternal characteristics and HEU infant outcomes. Second, we discuss growth outcomes, and set these findings within the wider context of other studies that have shown heterogeneous results. Third, we discuss the need for appropriate feeding strategies in HIV-exposed infants to ensure HIV-free survival and to reduce all-cause morbidity and mortality.

We propose a conceptual framework for poor outcomes in HEU infants and discuss the potential mechanisms underlying our key findings of infection susceptibility and growth failure, drawing also on other published data.

## The ZVITAMBO Trial

The ZVITAMBO trial was a randomized placebo-controlled trial of maternal and/or neonatal vitamin A to reduce HIV transmission and improve child mortality (1, 4). In brief, 14,110 postpartum mothers and their infants were enrolled within 96 h of delivery between November 1997 and January 2000 in Harare. Mothers and infants were eligible if neither had an acutely life-threatening condition; the infant was a singleton with birth weight ≥1500 g, and the mother planned to stay in Harare after delivery. A single large dose of vitamin A was given in a factorial design either to mother and infant, mother only, infant only, or neither. All but four infants started breast-feeding; at 6 months, 93% of infants were mixed breast-fed. The HIV status of mothers and infants was well characterized: at baseline, 9562 (67.8%) mothers were HIV-negative and 4495 (31.9%) HIV-positive; the remaining 53 mothers were HIV indeterminate. Of infants born to HIV-positive mothers, 381, 508, and 258 were infected *in utero*, intrapartum, and postnatally, respectively; 189 infants became HIV-infected, but the timing was uncertain; and 24 infants did not undergo PCR testing at any time, leaving 3135 live born infants who never had a PCR-positive test and were classified as HEU. Trial participants provided written informed consent for storage and use of data and samples for future, related studies. Overall, the trial found no effect of vitamin A on child mortality (1, 4) or on HIV transmission among HIV-exposed infants (1).

The ZVITAMBO trial offered a unique opportunity to study HEU infants for several key reasons: first, maternal and infant HIV status was well characterized throughout follow-up (see below); second, the HIV-unexposed comparison group was similar and contemporaneous; third, the majority of infants in each group had similar feeding patterns (mixed breast-feeding); and, fourth, all infants had access to a free “sick clinic,” allowing similar assessment of morbidity status between groups.

## Methods for Identifying HIV Infection

Studies investigating the outcomes of HEU infants need to ensure regular HIV testing of mothers and infants. Without repeat testing of mothers, those seroconverting during follow-up will be unidentified, meaning HIV-unexposed infant groups may be contaminated with HEU or HIV-infected infants; without repeat testing of infants, HEU infant groups may be contaminated with postnatally infected infants. In the ZVITAMBO trial, mothers were first tested for HIV at baseline; those testing HIV negative were retested at every subsequent blood draw to detect seroconversion. Infants born to mothers who remained HIV uninfected throughout follow-up were classified as HIV unexposed. Children born to HIV-positive mothers had samples stored at −70°C. At the end of the follow-up period, the last available sample from each child was tested for HIV; if this was negative, the child was classified as HEU. Various methods were used to ensure that the HEU group was not contaminated with postnatally infected infants, including censoring infants at the last negative HIV test if further testing was not conducted before the end of follow-up or infant death (as described below) (2, 5).

## Morbidity and Mortality of HEU Infants

### Morbidity

HIV-exposed uninfected infants in the ZVITAMBO trial had clear evidence of increased infectious morbidity compared to HIV-unexposed infants (5). HEU infants had 30% more sick clinic visits in the first year of life, peaking between 1 and 3 months of age, and 50% more hospitalizations within the first 28 days of life.

#### Sick Clinic Visits

The incidence of sick clinic visits among HEU infants was highest in the first 3 months of life, and remained significantly higher than for HIV-unexposed infants throughout infancy. The incidence rate ratios (IRR) for sick clinic visits were 1.2 (95% CI 1.1–1.4), 1.4 (1.3–1.5), 1.1 (1.1–1.2), and 1.1 (1.1–1.2) for 0–28, 29–91, 92–182, and 182–365 days, respectively. The most common illnesses among HEU infants were skin infections, lower respiratory tract infections, and oral thrush. Lower respiratory tract infections were particularly common in the first 3 months of life, with IRR of 1.6 (95% CI 1.1–2.3) and 1.5 (1.2–1.8) for 0–28 and 29–91 days, respectively (5).

#### Hospitalization

All-cause hospitalization was significantly higher in the first 28 days of life (IRR 1.5, 95% CI 1.2–2.0) among HEU compared to HIV-unexposed infants, with a trend toward increased all-cause hospitalization through 6 months of age. Hospitalization for malnutrition or diarrhea was common overall, but was not increased in HEU compared to HIV-unexposed infants, which may be due to the similar breast-feeding rates between groups. Increased risk of hospitalization for lower respiratory tract infections was particularly high in the first 28 days of life (IRR 2.7, 95% CI 1.6–4.7) (5).

### Mortality

HIV-exposed uninfected children had higher mortality than HIV-unexposed children through 2 years of age (2). The mortality difference between groups was twice as high during the first year (3.9-fold) compared to the second year of life (2.0-fold), highlighting infancy as a period of particularly high mortality, and suggesting an attenuation of mortality risk over time. The proportion of HEU infants who died by 30 days, 6 months, 1 and 2 years of age was 1.9% (95% CI 1.4–2.5), 6.0% (5.2–7.0), 7.4% (6.5–8.4) and 9.2% (8.1–10.5), respectively, compared to 0.7% (0.6–0.9), 1.6% (1.3–1.8), 1.9% (1.7–2.2) and 2.9% (2.5–3.5) of HIV-unexposed infants. Notably, the difference in mortality between groups was greater than the difference in morbidity; this may indicate greater severity of infections in HEU infants, as recently demonstrated in respiratory syncytial virus-associated lower respiratory tract infections in South Africa (6). The most common causes of death in HEU infants were acute respiratory infections (57.7%), diarrheal illness/dysentery (16.1%), malnutrition (13.3%), sepsis (6.0%), and meningitis (4.8%). Overall, the causes of death were similar between HIV-unexposed, HEU and HIV-infected infants.

### Sensitivity Analyses

Because of the possibility that the HEU infant group was contaminated with postnatally infected infants who died prior to testing HIV-positive, several sensitivity analyses were conducted. For morbidity, HEU infants were censored 42 days before their last HIV test (taking into account the window period for the test). The original analysis may in fact have underestimated morbidity among HEU infants, because the risk of hospitalization increased in sensitivity analyses, thereby adding confidence to the initial findings (5). For mortality, the sensitivity analyses included only those infants with at least one negative HIV test after 8 weeks of age; despite this, mortality remained 2.5-fold and 2.0-fold higher among HEU compared to HIV-unexposed infants by 12 and 24 months, respectively (2).

### Why Are HEU Infants at Risk of Infection and Death?

**Figure 1:**
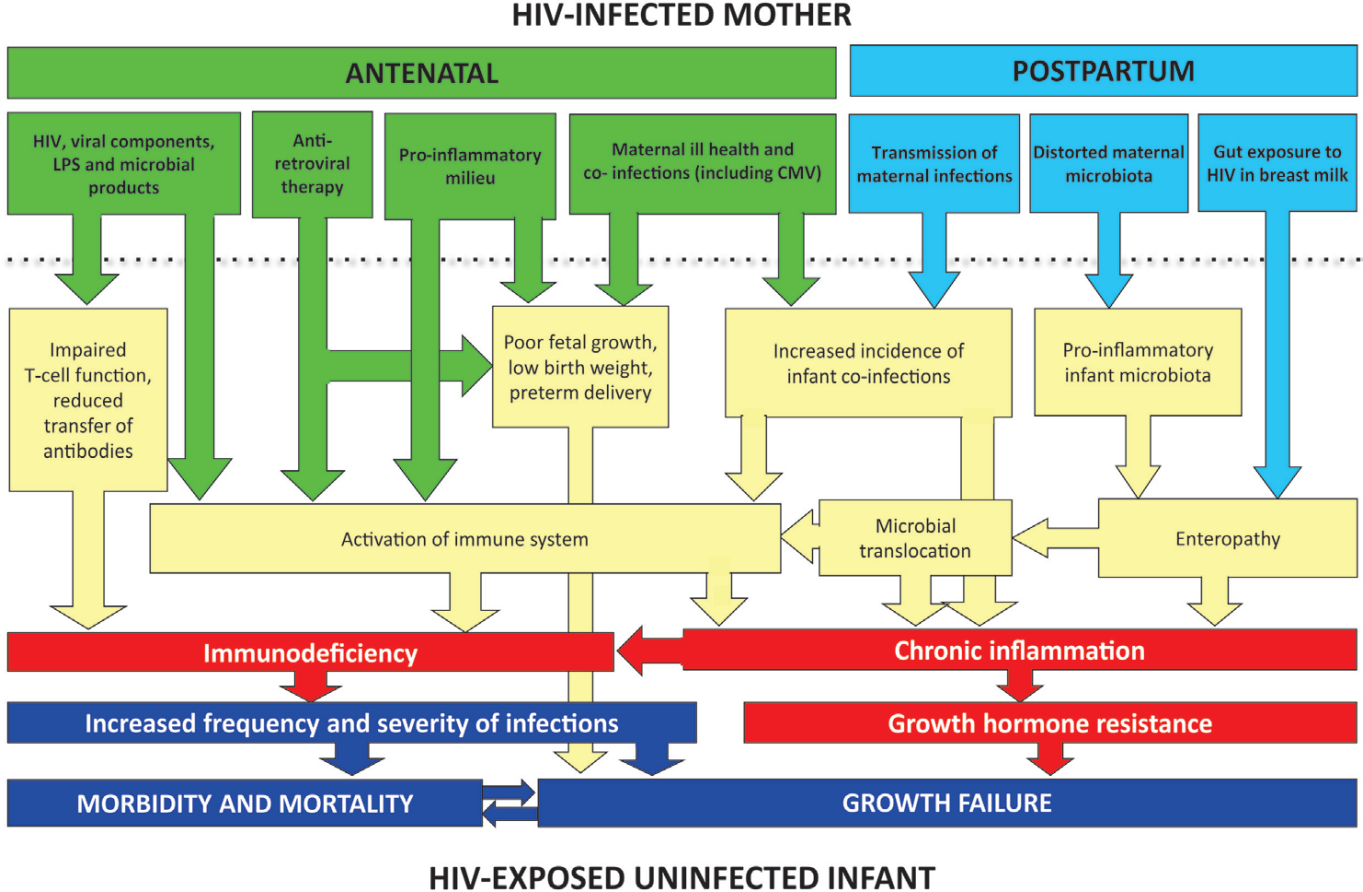
**Conceptual framework for poor clinical outcomes of HEU infants**. A combination of *in utero* and postnatal exposures may contribute to inflammation and immune activation in HEU infants. Immunodeficiency may be related directly to HIV exposure or may occur indirectly through reduced transplacental transfer of antibodies. Coinfections before and after birth (such as CMV and malaria) may also contribute to immune activation. Postnatally, exposure to HIV in breast milk may disrupt the intestinal barrier and lead to an enteropathy and microbial translocation. In non-breast-feeding infants, enteropathy may still be present secondary to abnormal assembly of the infant gut microbiota.

Here, we evaluate the potential underlying causes of morbidity and mortality in HEU infants, drawing on available evidence and plausible mechanisms from animal models and *in vitro* studies. In Figure 1, we propose a conceptual framework to explain infection susceptibility in HEU infants.

A growing body of evidence indicates that HEU infants have immunological abnormalities. First, studies show low concentrations of maternally derived antibody at birth (7–12). As newborns rely heavily on passive immunity before maturation of their own adaptive responses, this paucity of antibody may leave HEU infants at particular risk of infection. Second, there are numerous T-cell abnormalities: low CD4 count (13), high frequency of “double-negative” (CD4^–^/CD8^–^) T-cells (14, 15), and activated T-cell phenotypes (13, 14, 16–19) have all been well described. As T-cells are the primary target for HIV, it is perhaps unsurprising that they appear disproportionately affected in infants exposed to the virus. Third, HEU infants have elevated markers of immune activation and systemic inflammation (13, 14, 16–24).

Immune activation is an important cause of immune dysfunction in HIV-infected individuals, and its severity may be a better prognostic marker than HIV viral load (25). Animal models demonstrate the importance of chronic immune activation in growth failure and infection susceptibility. Transgenic mice that constitutively expressed CD70, leading to chronic T-cell stimulation, developed progressive naive T-cell depletion, weight loss, and premature death from *Pneumocystis jirovecii* pneumonia (26). Chronic immune activation in HEU infants may lead to infection susceptibility, and the resulting inflammation may further supress immune function. The causes of immune activation in HEU infants have not been well established; here, we speculate on plausible underlying causes (Figure 1).

#### Direct Exposure to the HIV Virus *In Utero* and the Influence of Maternal HIV Disease Severity

Fetal immune activation may result from direct exposure to HIV *in utero*; notably, HEU infants have evidence of HIV-specific T-cell responses (27, 28), suggestive of *in utero* sensitization. These responses are greater in infants born to mothers with high compared to low viral loads (29). Direct exposure to HIV or its components at a critical time of T-cell development *in utero* may contribute to the T-cell abnormalities described. HIV genomic material has been found in macrophages of the chorionic villus and in trophoblasts (30, 31). Components of HIV such as Nef have complex effects on the immune system, including CD4 depletion, activation, and apoptosis (32, 33). Furthermore, in a rodent model, Nef breaches placental barrier function and may enable HIV, other viral proteins and microbial products (see below) to cross the placenta (34), potentially exacerbating effects on the fetal immune system.

HIV-exposed uninfected infant morbidity and mortality outcomes in the ZVITAMBO trial were strongly influenced by maternal factors (Table 1) (2, 5). Infants born to mothers with more severe HIV disease (as assessed by maternal CD4 count) had higher rates of morbidity and mortality than those born to mothers with less severe HIV disease. Compared to HEU infants born to mothers with CD4 counts >400 cells/μL, those born to mothers with CD4 counts <200 cells/μL had 2.6-fold increased mortality by 2 years of age (95% CI 1.8–3.8). Increased morbidity risk remained until maternal CD4 counts were above 800 cells/μL. Oral candidiasis, an important indicator of immune function in the context of HIV, was particularly associated with maternal disease severity; compared to HIV-unexposed infants, HEU infants born to mothers with CD4 counts <200 cells/μL had an incidence rate ratio of oral thrush of 3.91 (95% CI 2.29–6.66), whereas those born to mothers with CD4 counts >800 cells/μL had an IRR of 1.91 (95% CI 1.02–3.58). The difference between these two HEU groups was statistically significant (*P* < 0.05).

**Table 1:** **Associations between maternal factors and HEU morbidity and mortality in the ZVITAMBO cohort**.

Maternal factor	Comparison group	Reference group	Outcome (95% confidence interval)
**Maternal disease severity and 2-year mortality of HEU infants (2)**			
Maternal CD4 count (cells/μL)	<200	≥400	HR 2.62 (1.8–3.8)^a^
	200–400	≥400	HR 1.26 (0.9–1.5)
Hemoglobin (g/L)	<70	≥70	HR 3.79 (2.06–6.97)^a^
Maternal survival (at 12 months after delivery)	Died	Survived	HR 2.68 (1.86–3.87)^a^
**Maternal social status and 2-year mortality of HEU infants (2)**			
Marital status	Single	Married/cohabiting	HR 2.55 (1.29–5.06)^a^
	Widowed	Married/cohabiting	HR 1.97 (1.25–3.12)^a^
Household income (US $)	<1.20	≥2.40	HR 1.87 (1.28–2.73)^a^
	1.20–2.40	≥2.40	HR 1.30 (0.91–1.84)
**Maternal disease severity and 2-year sick clinic visit in HEU infants, compared to HUU infants (5)**			
Maternal CD4 count (cells/μL)	<200	HUU infants	IRR 1.33 (1.17–1.50)^b,c^
	200–499	HUU infants	IRR 1.24 (1.17–1.32)^b,c^
	500–799	HUU infants	IRR 1.11 (1.08–1.27)^b^
	≥800	HUU infants	IRR 1.02 (0.89–1.16)

*Based on data presented in Marinda et al. (2) and Koyanagi et al. (5)*.

*HR, hazard ratio; IRR, incidence rate ratio; HEU, HIV-exposed uninfected; HUU, HIV-unexposed uninfected*.

*Hazard ratios (HR) calculated using Cox proportional hazard models*.

*Incident rate ratios (IRR) calculated using negative binomial with the HUU infants as the reference group*.

*^a^Significant difference between comparison groups (HEU vs. HEU)*.

*^b^Significant difference between comparison groups (HEU vs. HUU)*.

*^c^Significant difference between that group and HEU infants born to mothers with CD4 count ≥800 cells/μL (HEU vs. HEU)*.

The relative influences of maternal viremia (as assessed by viral load) and maternal immune suppression (as assessed by CD4 count) on HEU outcomes should be determined in future studies; this may help to establish the drivers of infection susceptibility, particularly in the current ART era.

#### Increased Exposure to Coinfections

Coinfections such as cytomegalovirus (CMV) are more prevalent in HIV-infected compared to HIV-uninfected pregnant women (35, 36), and could contribute to immune activation in their offspring both before (37) and after (38–40) birth. Infants born to HIV-infected mothers have a very high frequency of congenital CMV infection (11–29%) (36, 41, 42), and postnatal infection is almost ubiquitous during infancy in sub-Saharan Africa. There is an overlap between the effects of CMV infection and HIV exposure in infancy, including growth failure (43) and mortality (43, 44). For example, HIV-exposed infants with pneumonia in South Africa had 4.3-fold higher frequency of CMV viremia compared to asymptomatic HIV-exposed infants after adjusting for infant HIV infection (95% CI 2.6–7.0) (45).

There are plausible reasons why CMV acquisition may lead to immune dysfunction. First, in order to evade the immune system and promote latency, CMV has evolved multiple immunomodulatory properties to downregulate the human immune system (46, 47). Therefore, CMV infection in early life could be associated with increased susceptibility to other childhood infections. Second, evidence from humans and from murine models suggests that CMV causes immune activation. Studies report associations between primary and latent CMV infection and immune activation in adults; (48) a bias toward pro-inflammatory and Th17-polarized cytokines in the placenta and amniotic fluid during maternal CMV infection; (49) a lower regulatory T-cell (Treg)/Th17 ratio in CMV-infected children; and an impact of CMV coinfection on immune activation in HIV-infected individuals (50). Th17-polarized cytokines are associated with increased inflammation in response to viral infections, and it has been suggested that this may increase mortality (51). Furthermore, inflammation associated with CMV carrier status may affect responses to vaccinations (52). Third, CMV is acquired either *in utero* or early in infancy in sub-Saharan Africa and typically induces large magnitude immune responses. Infants with congenital CMV infection have evidence of considerable expansions in γδ T-cells (53), NK cells (54), and conventional αβ T-cells with a highly differentiated phenotype (55). Whether primary CMV infection at a critical time of immune development causes immunomodulation in HEU infants, and whether this alters infection susceptibility or mortality, has not been well addressed to date. We hypothesize that primary CMV infection in HEU infants results in inflammation and distortion of the immune system, leading to increased infection susceptibility, but further studies are required.

Pharmacological interventions to prevent CMV transmission to HEU infants have been considered a potentially important intervention but have so far reported unfavorable results. A Kenyan trial, in which women were randomized to 12 months of valaciclovir or placebo from 34 gestational weeks, did not reduce CMV transmission to HEU infants (56), and maternal nelfinavir for at least the last 4 weeks of pregnancy did not reduce congenital CMV in HEU infants in the USA (57). Formula feeding of HEU infants was associated with a lower incidence of CMV infection by 1 year of age in Kenya (58), highlighting breast-feeding as a major route of CMV transmission. However, the risks of formula feeding make it an impractical intervention to reduce CMV in developing countries.

#### Maternal Microbial Translocation

Microbial translocation causes immune activation in HIV infection (59), and is a key distinction between HIV infection in humans and non-pathogenic simian immunodeficiency virus (SIV) infection in sooty mangabeys (60). Lipopolysaccharide (LPS) and other bacterial components from intestinal microbes are able to cross a leaky gut barrier as a direct result of HIV exposure, activating immune cells in the systemic circulation; increased LPS, as a marker of translocation, has been directly associated with innate and adaptive immune activation in HIV infection (59). HIV-infected pregnant women have higher levels of circulating soluble CD14 (sCD14) and LPS-binding protein than HIV-uninfected women, suggesting that microbial translocation occurs throughout pregnancy; first trimester sCD14 levels were independently associated with preterm birth (61) in multivariate analyses. In an animal model (62), subclinical infection with murine gammaherpesvirus 68 sensitizes pregnant mice to a greater cytokine response to LPS, suggesting that viral infections have potential to amplify the impact of LPS exposure during pregnancy. Maternal microbial translocation in HIV-affected pregnancies may plausibly contribute to immune activation in HEU infants: LPS can cross the placental barrier in mice (63), meaning that translocated maternal LPS could potentially activate fetal immune cells, particularly in the context of Nef-mediated placental barrier dysfunction (34). Whether LPS or other microbial products cross the placental barrier in HEU infants and contribute to immune activation has yet to be confirmed.

Notably, microbial translocation, and its associated immune activation, often persists in HIV-infected individuals despite ART (64–67). If this mechanism is related to immune activation in HEU infants, HEU infants may continue to be at risk of infection susceptibility despite maternal viral suppression throughout pregnancy.

#### Postnatal Exposure to HIV

Postnatal contact between HIV and the gut epithelium during breast-feeding may damage the mucosal barrier, enabling infant microbial translocation (68), although we recently showed that plasma levels of intestinal fatty acid binding protein, one marker of small intestinal villous damage, were similar between HIV-exposed and HIV-unexposed infants in the ZVITAMBO trial, and between HIV-exposed infants who did and did not acquire postnatal HIV infection through breast milk.^1^ In South Africa, greater exclusivity of breast-feeding was associated with less gut inflammation; (69) among infants recruited to the BAN trial, plasma LPS, a marker of microbial translocation, was higher after compared to before weaning (70).

#### Socioeconomic Factors

Social and economic factors are also likely to be important in infection susceptibility among HEU infants, including parental health and parental care-taking capacities; socioeconomic status; and pathogen exposure in the home environment, although a detailed discussion of these remains outside the scope of this Review. In the ZVITAMBO trial, morbidity and mortality outcomes were associated with family social factors, including parental relationship stability and household income (Table 1).

#### Postnatal Vitamin A

Vitamin A supplementation had no effect on HIV transmission in the ZVITAMBO trial, whether it was given to mothers, infants, or both. Furthermore, maternal and/or infant vitamin A had no overall effect on child mortality. However, in subgroup analyses, it was found that vitamin A had heterogeneous effects. Maternal or neonatal vitamin A had no effect on infants who acquired HIV *in utero*, but neonatal vitamin A did reduce mortality in those infected around the time of birth. In infants who were uninfected at 6 weeks of age (a group that included HEU and infants who later became postnatally infected), vitamin A was associated with higher 24-month mortality compared to placebo (Table 2). On sensitivity analysis, it appeared that the majority of those who died were HIV-infected before death. It is plausible therefore that vitamin A prior to HIV infection hastened disease progression when infants were subsequently infected through breast milk transmission of HIV (1).

**Table 2:** **Associations between maternal and infant vitamin A exposure and 24-month mortality in HIV-exposed infants remaining HIV PCR negative at 6 weeks (*n* = 2876) in ZVITAMBO**.

		Infant randomization	
		Vitamin A	Placebo
Mother randomization	Vitamin A	2.05 (1.14–3.67) *P* = 0.02	1.82 (0.99–3.31) *P* = 0.05
	Placebo	1.89 (1.05–3.40) *P* = 0.03	1.00
		**Infant randomization**	
		**Vitamin A**	**Placebo**
Mother randomization	Vitamin A	1.41 (0.97–2.05) *P* = 0.07	1.00
	Placebo		
		**Infant randomization**	
		**Vitamin A**	**Placebso**
Mother randomization	Vitamin A	1.33 (0.92–1.92) *P* = 0.14	
	Placebo	1.00	

*Based on data presented in Humphrey et al. (1)*.

*Adjusted hazard ratios (95% confidence intervals) calculated by Cox proportional hazard models with the following covariates – maternal mid-upper arm circumference, maternal death, maternal CD4 count, maternal hemoglobin, and maternal marital status*.

#### Future Studies

Future studies should focus on better characterizing the nature and drivers of immunodeficiency in HEU infants and the relationship between immune ontogeny and clinical outcomes in this group. Whether immune dysfunction among HEU infants can be prevented or ameliorated with use of maternal ART or with other interventions remains unclear. However, immune activation in HIV-infected adults persists despite ART (64–67), and there is some evidence that ART exposure may actually worsen immune activation in HEU infants: in South Africa, T-cell activation at birth was unexpectedly higher among HEU infants exposed to antenatal and postnatal nevirapine compared to those unexposed to ART, perhaps due to an activating effect of nevirapine either on HIV-infected or bystander cells (71).

Studies of prospective cohorts are needed to evaluate the relationship between markers of immune dysfunction, such as immune activation, and infection susceptibility in HEU infants. Furthermore, studies comparing infection susceptibility and mortality outcomes in HEU and HIV-unexposed infants in context of suppressive maternal ART are required, in order to determine if the differences seen in the ZVITAMBO trial remain modern era. Although studies have been undertaken in the era of short periods of ART for PMTCT, there have so far been no studies undertaken in the setting of fully suppressive maternal ART throughout pregnancy, which is now the standard of care.

## Growth of HEU Infants in the ZVITAMBO Trial

Growth failure is common in sub-Saharan Africa and is associated with childhood mortality (72). Poor growth has been well described in HIV-infected infants and children, but the effect of maternal HIV on the growth of HEU infants is less clear. Although the majority of cohorts across sub-Saharan Africa have shown trends toward poorer growth among HEU infants, many results do not reach statistical significance, potentially due to small numbers of children (3). We recently showed in the ZVITAMBO trial that HEU infants were more likely to be born premature and small-for-gestational age (SGA) than HIV-unexposed infants. HEU infants had evidence of growth failure at birth (2), and mean length-for-age and weight-for-age *Z*-scores remained lower among HEU compared to HIV-unexposed infants throughout 2 years of follow-up. The differences in growth between HEU and HIV-unexposed infants peaked at 6 weeks of age, when HEU infants were 25% more likely to be stunted, 55% more likely to be underweight, and 58% more likely to be wasted; these differences in stunting, underweight, and wasting persisted until 1 year of age.^2^ Compared to HIV-unexposed infants, HEU infants also had poorer head growth in the first year of life,^3^ although the influence of such a finding on neurodevelopmental outcomes remains uncertain.

To ensure robust growth outcome results, HEU infants were censored from analyses at their last negative HIV test.

### Why Are HEU Infants at Risk of Growth Failure?

A number of mechanisms may underlie the association between maternal HIV infection and poor infant growth. Maternal immune activation may lead to pro-inflammatory vascular damage resulting in reduced placental blood supply (73) or increased placental inflammation and chorio-amnionitis; (74) each may lead to poor fetal growth. The high burden of coinfections in HIV-infected women may also influence growth of their offspring; for example, coinfection with HIV and malaria causes SGA (75, 76), which may be due to modifications in the placental cytokine environment (77). Congenital CMV infection causes poor fetal growth (43), and this effect may be more pronounced in infants of HIV-infected mothers.

#### Inflammation

Inflammation may also be a key driver of growth failure. In a recent sub-study of HIV-unexposed infants from the ZVITAMBO trial, we found linear growth failure was related both to acute illness and to chronic inflammation, with both clinical and subclinical disease associated with suppression of the growth hormone axis (78, 79). At 6 weeks of age, HEU infants in ZVITAMBO had higher C-reactive protein (CRP) than HIV-unexposed infants^1^; it is therefore plausible that the growth failure seen in HEU infants is related to higher levels of systemic inflammation leading to reduced insulin-like growth factor 1 (79); however, further data are needed to test this hypothesis.

#### Microbiota and Enteropathy

The intestinal microbiota is emerging as a key contributor to healthy postnatal growth, and a series of recent studies (80–82) has established that maturational defects in the composition and function of the microbiota underlie malnutrition in developing countries. An inflammatory pathology of the small intestine, termed environmental enteric dysfunction, is a potentially important cause of stunting among young children in developing countries (83, 84) and may be related to the configuration and function of the microbiota (85). The early life infant microbiota is founded following vertical transmission from the mother (86), so a distorted maternal microbiota in the context of HIV infection (87, 88) may lead to abnormal assembly of the microbiota in HEU infants, which could plausibly drive growth failure through subclinical intestinal damage and inflammation. However, to our knowledge, this has not been investigated to date. Greater intestinal inflammation and increased microbial translocation could drive systemic inflammation and immune activation in HEU infants, and could therefore contribute to both growth restriction and infection susceptibility. Notably, there are higher rates of stunting in HEU infants affected by diarrhea compared to those without diarrhea (89). Differences in microbiota composition and small intestinal pathology between HEU and HIV-unexposed infants warrant further exploration, as these processes are potentially amenable to gut-focused therapy.

#### Intrauterine Growth Restriction and Preterm Birth

It has been estimated that around 20% of stunting has fetal origins (90). In HEU infants born at term in the ZVITAMBO trial, SGA and length-for-age *Z*-score at birth were closely associated with stunting, highlighting intrauterine growth and development as a key contributor to future growth potential of HEU infants. Conversely, preterm birth without associated SGA was not associated with poorer growth trends across the first 12 months of life^2^.

#### Maternal Disease Severity

In contrast to morbidity and mortality, growth among HEU infants in ZVITAMBO was not associated with maternal disease severity (as determined by CD4 count at birth). This is also in contrast to studies from other countries that showed poorer fetal (91) and postnatal (92, 93) growth in HEU infants born to mothers with greater HIV disease severity. A recent study from rural Uganda highlights the importance of maternal nutritional status and HEU infant growth; HIV mothers with poor weight gain throughout pregnancy were more likely to have preterm and/or low-birth-weight HEU infants.

#### Antiretroviral Therapy

As infant growth was not associated with maternal disease severity in ZVITAMBO, virological suppression and immune reconstitution on ART may not necessarily improve growth outcomes. A Ugandan study undertaken in the context of maternal ART demonstrated a non-significant trend toward higher rates of stunting among HEU compared to HIV-unexposed infants [12-month adjusted odds ratio (OR) 1.55, 95% CI 0.92–2.61] (94), but this result may have been limited by a relatively small number of HEU infants included in the study. Differences in ponderal growth have not been found in most studies from the ART era (15, 94–98), although one Ugandan study reported an adjusted OR for wasting of 3.29 at median 5.2 months of age (*P* = 0.02) among HEU compared to HIV-unexposed infants (99). As wasting is often associated with acute illness, it is plausible that ART-related improvements in HEU infant immune function may reduce infection susceptibility and improve weight gain.

However, exposure to ART itself throughout fetal development, and choice of maternal ART regimen, may influence differences in birth outcomes and growth. It is becoming clear that maternal ART is associated with an elevated risk of adverse birth outcomes (100). In the Promoting Maternal–Infant Survival Everywhere (PROMISE) trial (101), women were randomized to lopinavir/ritonavir-based ART or zidovudine alone with intrapartum single-dose nevirapine; although mother-to-child tranmission was significantly lower in the combination ART arm, preterm delivery was significantly higher (20.5 vs. 13.1%). In some (102) studies, but not others (103), protease inhibitor-based ART has been particularly associated with risk of preterm birth (104). In most of sub-Saharan Africa, efavrienz-based ART is the standard regimen used in pregnancy; however, there is still evidence of increased preterm delivery and SGA (105, 106) following exposure to non-nucleoside reverse transcriptase inhibitor (NNRTI)-based regimens. Growth deficits associated with ART exposure that are evident at birth may persist and contribute to postnatal growth failure. For example, in Botswana, birth LAZ, WAZ, and WLZ were each significantly lower among infants exposed to triple ART compared to zidovudine monotherapy; LAZ remained significantly lower through 6 months of follow-up (107).

#### Socioeconomic Factors

In the ZVITAMBO trial, wasting and underweight were more frequent in HEU infants born to mothers with primary compared to A level education. Interestingly, this educational difference was apparent despite very high levels of literacy across the study population.

Taken together, there are several plausible mechanisms that could lead to growth failure in HEU infants (Figure 1), although further clinical and laboratory studies are required to dissect these mechanistic pathways further. Notably, data from the ZVITAMBO trial are unable to determine the causal association between morbidity and growth failure; poor growth may result from a higher incidence of infections, or may be itself be a cause of infection susceptibility.

## Feeding HEU Infants

Targeting HIV-infected mothers and their exposed children with appropriate clinical and nutritional interventions is critical to improve survival. Until the availability of highly effective ART interventions for HIV-positive mothers and their infants, breast-feeding caused more than 200,000 new cases of pediatric HIV globally each year (108), but also prevented millions of infant deaths [one Ugandan trial reported that HEU infant mortality was over sixfold lower in infants who breast-fed for longer than 6 months compared to those who breast-fed for a shorter duration (109)]. Infant feeding therefore became one of the most profound dilemmas of the HIV epidemic.

The ZVITAMBO trial provided strong evidence for the association between exclusive breast-feeding (EBF) and reduced risk of postnatal HIV transmission (110). Among HIV-positive mothers, early EBF (feeding only breast milk) was associated with a 75% reduction in breast-feeding-associated HIV transmission by 6 months of age compared with early mixed breast-feeding (feeding both breast milk and non-breast milk liquid or solid foods). Compared to early EBF, early mixed breast-feeding was associated with 4.03-fold (95% CI 0.98–16.61), 3.79-fold (1.40–10.29), and 2.60-fold (1.21–5.55) greater risk of postnatal HIV transmission at 6, 12, and 18 months, respectively (110). This finding was due to nesting a “natural experiment” within the ZVITAMBO trial: 8 months after the trial was launched, WHO released policy that HIV testing and counseling should be available to all antenatal women, allowing mothers to make informed decisions about infant feeding (111). Importantly, the policy recommended early EBF and continued breast-feeding for women of unknown HIV status (the majority of women enrolled into the trial chose against learning their HIV status). In response, ZVITAMBO introduced an infant feeding intervention to support known HIV-positive women to make empowered choices about infant feeding, and to promote “safer breast-feeding” among HIV-negative mothers and those unwilling to learn their HIV status (early EBF; safe sex to avoid new HIV infections; prompt treatment of, and optimal techniques to reduce, breast problems) (112). Contact with this intervention was the strongest predictor of EBF in ZVITAMBO: EBF rates increased and postnatal HIV transmission rates declined with each additional exposure to the intervention (112).

### Why Is Exclusive Breast-feeding Associated with Better HIV-exposed Infant Outcomes?

The underlying reasons for the protective effect of EBF on PMTCT remain uncertain (113). We hypothesize that early introduction of non-milk fluids and solid food, as is the cultural norm in Zimbabwe (112), increases intestinal inflammation due to modulation of the microbiota (114) or introduction of pathogenic bacteria (115). Intestinal inflammation may impair gut integrity and increase the pool of activated intestinal CD4 cells that are targeted by the virus. In the BAN trial, infants had higher markers of microbial translocation (LPS) after weaning than before, and pre-transmission LPS levels were a predictor of subsequent infection (70). It has alternatively been proposed that the association between EBF and reduced breast milk transmission is due to reverse causality – that women in better health, who are less likely to transmit HIV, are also more likely to exclusively breast-feed.

### Breast-feeding Interventions

Although continued breast-feeding after 6 months is likely to provide the greatest chance of survival for the majority of infants living in developing countries, this policy has proved difficult to implement. However, recent experience in Zimbabwe indicates that interventions targeting specific contextual barriers may be successful in increasing rates of EBF to 6 months (116–118). HIV-exposed infants may benefit from targeted EBF promotion; contact with healthcare professionals at birth or during early infant diagnosis at 6 weeks of age could be an opportunity to empower HIV-infected mothers to exclusively breast-feed. As contact with a counseling program was the strongest predictor of EBF in the ZVITAMBO trial (112), such programs may be paramount to empower women and improve the health of HEU infants.

## Summary

HIV-exposed uninfected infants in the ZVITAMBO trial had poorer health outcomes than HIV-unexposed infants. Hospitalization was 50% more frequent among HEU infants in the first month of life, and mortality rates were higher over the 24-month follow-up period. Morbidity and mortality outcomes were associated with maternal disease severity and social factors, including parental relationship stability and household income (Table 1). HEU infants were at higher risk of stunting, wasting, and underweight than HIV-unexposed infants, although maternal disease severity was not associated with growth outcomes.

We propose that immune activation and inflammation and may be key drivers of both infection susceptibility and growth failure in HEU infants. Notably, baseline CRP was higher in HEU compared to HIV-unexposed infants at 6 weeks of age, and was still elevated at 6 months of age. Our conceptual framework highlights a number of key pathways that may drive inflammation and immune activation, including maternal HIV itself (*in utero* and postnatally); coinfections (such as CMV and malaria); and a distorted gut microbiota (which may be acquired from the HIV-infected mother). Future work will aim to elucidate and describe pathways leading to poor health outcomes of HEU infants.

## Conclusion

Infants recruited to the ZVITAMBO trial have contributed to our understanding of the HEU population. Future studies should draw on these and other results in order to determine the causes of infection susceptibility and growth failure, and determine the impact of ART and cotrimoxazole on outcomes of this vulnerable group of infants.

## Author Contributions

CE wrote the first draft of the manuscript, which was critically reviewed and revised by JH, RN, and AP. JH designed and recruited to the original ZVITAMBO trial. RN was a coinvestigator on the original ZVITAMBO trial.

## Conflict of Interest Statement

The authors declare that the research was conducted in the absence of any commercial or financial relationships that could be construed as a potential conflict of interest. CE is funded by the National Institute for Health Research. AJP is funded by the Wellcome Trust (108065/Z/15/Z).

## Footnotes

^1^ Prendergast AJ, Chasekwa B, Rukobo S, Govha M, Mutasa K, Ntozini R, et al. Intestinal damage and immune activation in HIV-exposed and HIV-infected Zimbabwean infants (submitted 2016).

^2^Omoni AO, Ntozini R, Evans C, Prendergast AJ, Moulton LH, Christian PS, et al. Child growth according to maternal and child HIV status in Zimbabwe (submitted 2016).

^3^Evans C, Chasekwa B, Ntozini R, Humphrey JH, Prendergast AJ. Head circumferences of children born to HIV-infected and HIV-uninfected mothers in Zimbabwe during the pre-ART era (submitted 2016).
